# Novel Green Nanotechnologies Applied in Environmental Protection and Health

**DOI:** 10.3390/ma15155297

**Published:** 2022-08-01

**Authors:** Marcela-Elisabeta Barbinta-Patrascu, Nicoleta Badea, Irina Zgura

**Affiliations:** 1 Department of Electricity, Solid-State Physics and Biophysics, Faculty of Physics, University of Bucharest, 405 Atomistilor Street, 077125 Magurele, Romania; 2 General Chemistry Department, Faculty of Chemical Engineering and Biotechnology, University “Politehnica” of Bucharest, 1–7 Polizu Street, 011061 Bucharest, Romania; nicoleta.badea@upb.ro; 3 National Institute of Materials Physics, Atomistilor 405A, 077125 Magurele, Romania; irina.zgura@infim.ro

Today, humanity is facing serious problems due to the environmental pollution. Several tons of plastic or industrial wastes are dumped randomly in nature, polluting waters and soils, thus creating many health problems for people and all living things. To keep the Earth clean, we need to adopt eco-friendly strategies sustaining human and environmental health. Green nanotechnology—the science of the future—can help to prevent future environmental problems and improve the general quality of life and human well-being. In addition, bioinspiration and biomimetics became new trends in green nanotechnology for the “green” development of multifunctional materials with potential applications in the biomedical field and in environmental protection [1,2,3].

It is known that, since ancient times, most sources of natural substances with healing effects are of plant origin. Plants contain a wide range of substances, which have been used for millennia in traditional medicine [4,5]. Researchers have tried to replace synthetic products with natural products, such as plant extracts, because herbal extracts/essential oils contain compounds with fungicidal properties [6]. These compounds have several advantages. They have fewer side effects for humans and are more environmentally friendly and have lower production costs; in some cases, their antimicrobial properties may be superior to synthetic products [7]. Plants are preferred in the synthesis of metal nanoparticles due to their availability, low cost, abundance in nature, recyclability of vegetable residues and thus production costs are minimized [8,9,10]. The phyto-materials have attracted more and more attention to the scientific world because they combine the benefits of plants with other components, resulting in materials with improved properties and extended applicability. A wide variety of chemical compounds with different structures and properties can be produced from plants. These include phenols, alkaloids, saponins, terpenoids, flavonoids, etc. In recent years, these phyto-substances have been the subject of numerous *in vitro* and *in vivo* tests to determine their effectiveness as antimicrobial agents against pathogenic bacteria, fungi and viruses, or as antioxidant and anti-cancer agents.

This special issue “Novel Green Nanotechnologies Applied in Environmental Protection and Health” aimed to highlight many aspects related to “green” approaches to design innovative materials/systems, and eco-friendly strategies for applications in Environmental Protection and Health. Interesting topics are proposed. By using natural resources and recycling food and vegetal wastes, and also the bioinspiration and biomimetics for development of novel materials, the environment pollution can be avoided, thus preventing severe problems affecting human health.

This Special Issue kindly invites authors to contribute with original research articles and review papers describing novel green nanotechnologies applied to design eco-friendly materials, by exploiting natural resources and recycling food and vegetal wastes, and converting them into valuable materials with applications in various fields.

Potential topics include, but are not limited to the following:

- Eco-nano technologies and advanced materials;
- Environmental and biomedical applications of “green” nanomaterials;
- Eco-friendly strategies for development of (bio)composite/(bio)polymeric materials;
- Biomedical applications of nanoparticles/materials;
- Nanoparticles for drug delivery systems;
- Biological activity of nanoparticles;
- “Green” materials and technologies for electronics;
- Bioinspiration and biomimetics for development of novel materials;
- Bioplastics;
- Eco-friendly packaging materials;
- Biopesticides;
- Photocatalysis;
- Wastewater treatment;
- Eco-Friendly Corrosion Inhibitors.
